# Evaluating the Impact of Scanning Factors on Ultrasound Imaging for Predicting Semen Quality in Boars

**DOI:** 10.3390/ani16081131

**Published:** 2026-04-08

**Authors:** Shihong Yang, Yijian Huang, Jeremy Howard, Vance Brown, Chun-Peng James Chen

**Affiliations:** 1 School of Animal Sciences, Virginia Polytechnic Institute and State University, Blacksburg, VA 24061, USA; shihong@vt.edu; 2 Smithfield Premium Genetics, Smithfield Foods, Rose Hill, NC 28458, USA; yhuang@smithfield.com (Y.H.); jhoward@smithfield.com (J.H.); vbrown@minitube.com (V.B.)

**Keywords:** computer vision, feature extraction, precision livestock farming, semen quality, ultrasound imaging

## Abstract

Early prediction of semen quality in young boars is crucial to reduce operational costs associated with low-productivity boars at boar studs, where high genetic merit is key. B-ultrasound imaging, a non-invasive method using echo signals to visualize tissue density, has been evaluated as a potential tool for this purpose. Stronger echoes indicate denser tissues, such as the seminiferous tubules responsible for sperm production. This study focuses on investigating the predictive power of using B-ultrasound imaging from boars around 6 to 9 months old to predict semen quality over the next six months in industrial settings. Several scanning factors were considered, including image brightness, imaging area of the testicle, and the imaging angle of the probe. The studied dataset included 1417 images and 3254 semen records from 107 boars. Results showed that the model’s performance was significantly influenced by the imaging area, the angle of the testicle, and the pixel brightness of the image without being standardized. While the accuracy of B-ultrasound imaging is not yet sufficient to replace traditional assessments, this study highlights key features in testicular images that may significantly impact model predictions, providing practical guidance for leveraging B-ultrasound imaging in predicting semen quality in young boars.

## Simple Summary

Early identification of high-quality breeding boars is essential for reducing costs and improving efficiency on pig farms. This study investigated whether ultrasound imaging as a safe and non-invasive technology could predict the future semen quality of young boars aged six to nine months. By analyzing a large dataset of ultrasound images and breeding records, we found that specific factors during the scanning process significantly affect the accuracy of these predictions. These factors include the specific area of the testicle being scanned, the angle of the imaging probe, and the natural brightness of the images. While this ultrasound method is not yet accurate enough to fully replace traditional lab tests, our research identifies the most important imaging features for making reliable predictions. These findings provide practical guidance for farmers and researchers to better use ultrasound technology. This helps them select the best breeding animals earlier and contributes to more sustainable livestock management.

## 1. Introduction

Semen quality is crucial in breeding programs as it supports the transfer of desirable traits and boosts productivity through increased pregnancy rates and litter sizes [1,2]. Beyond basic health, it is essential for selecting boars that strengthen traits like muscle growth and structural resilience [3]. Recent molecular advances have further refined evaluation by ensuring robust immunity and growth characteristics are reliably passed on [4]. Additionally, improved cryopreservation supports consistent year-round productivity and simplified herd management [5].

In traditional management, boars are selected by 4–5 months, trained for collection during an isolation period, and enter regular production at around 8–9 months [6]. Throughout this process, semen quality is closely monitored using traditional metrics including motility, morphology, and concentration, alongside emerging molecular markers like heat shock proteins and oxidative stress markers [7,8]. To validate these laboratory findings, in vivo data like fertilization rates are used to improve predictive precision.

Artificial Intelligence (AI) has transformed medical imaging by enhancing diagnostic efficiency and identifying abnormalities that may escape human interpretation [9]. In veterinary medicine, AI supports precision livestock farming (PLF) by enabling real-time monitoring and earlier intervention [10]. While Deep Learning (DL) has been applied to various animal species, its use in boar testicular imaging remains underexplored. B-ultrasound imaging offers a non-invasive way to assess reproductive potential early, allowing breeders to identify high-potential boars before maturity [11]. This reduces the cost of raising low-fertility animals and minimizes the need for repeated testing.

In addition to visual assessment, quantitative echotexture analysis has been applied in reproductive studies of domestic species such as bulls and rams [12,13]. These approaches, using image-analysis tools such as ImageJ and ECOTEXT^®^, extract biologically relevant parameters from ultrasound images, including pixel intensity distributions and structural features such as seminiferous tubule density and lumen area [12]. These features are associated with tissue composition and spermatogenic activity, providing a biologically interpretable basis for evaluating testicular function [12]. Traditional methods rely on predefined features and may be influenced by operator-dependent procedures, which can limit scalability in commercial settings. In contrast, the data-driven approach used in this study may capture complex spatial relationships and broader anatomical context that are not explicitly represented by predefined echotexture features.

To overcome these limitations, deep learning (DL) approaches have been increasingly explored to automatically learn relevant features from ultrasound images without predefined descriptors. DL models, such as YOLOv11, enhance ultrasound analysis by identifying subtle variations in tissue texture, size, and shape [14]. YOLOv11’s multi-scale feature extraction is particularly effective at capturing density and brightness variations influenced by physiological structures [15]. However, the transition from experimental settings to real-world commercial production remains challenging due to the scarcity of large-scale datasets, inconsistent image quality, and the labor-intensive nature of expert annotation [16,17].

This study bridges the gap between AI innovation and practical swine production by evaluating the factors influencing DL-based semen quality prediction using data obtained from a commercial farm under standardized imaging conditions. The model is trained on early testicular ultrasound images obtained from boars aged 6–9 months to classify individuals into two categories at maturity: boars with consistently high semen quality and those showing underperforming semen production based on repeated semen collection outcomes during the evaluation period. By utilizing standardized field data, we ensure the practical applicability of our findings for precision breeding programs. The study systematically evaluates how four key factors impact reliability: image region, ultrasound probe angle, model type, and augmentation, providing essential guidance for the refinement of AI-identified features in large-scale livestock management.

## 2. Materials and Methods

This study was conducted on a commercial pig farm in North Carolina, United States. Standard operating procedures were strictly followed by animal caretakers for all animal handling processes and procedures in accordance with both Smithfield Animal care policies and National Pork Quality Assurance Plus (PQA+) Program. The farm maintains an active certification through the PQA+ program (reference number: 00DTE2H).

### 2.1. Animal Husbandry and Data Collection

**Figure 1:**
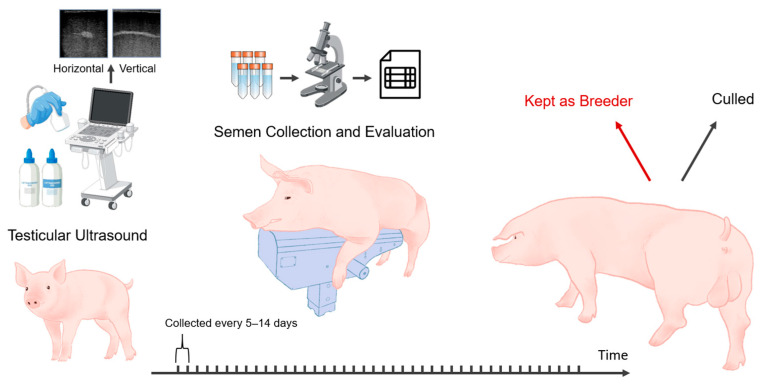
Ultrasound images were collected from 107 boars at 6–9 months of age, followed by semen collection and evaluation every 5–14 days for about a year to identify high-fertility potential breeders.

This study included 107 young boars, all born in 2021, with birth dates ranging from March to June. The boars were predominantly of the Duroc breed, with a single exception being a Large White. Given that only one individual belonged to the Large White breed (<1% of the dataset), it was retained in the analysis, as its inclusion was unlikely to introduce systematic bias to the model. Each animal was assigned a unique Boar ID to facilitate tracking and differentiation throughout the study. This Boar ID was also used for matching the ultrasound images with the corresponding semen quality and quantity assessment data. As depicted in Figure 1, the study began in mid-December 2021 with technicians completing a one-day ultrasound imaging session of all boars’ testicles when the boars were around 6 to 9 months old. This session resulted in 1417 ultrasound images, with each boar contributing between 9 and 17 images (median of 12 images per boar). Following the imaging, semen collection and evaluation took place throughout 2022 at intervals of 5 to 14 days, continuing for up to 50 weeks. Semen quality classification was based on repeated measurements collected over this extended period, thereby reflecting cumulative reproductive performance rather than single time-point observations. Although minor variations occurred due to farm management practices, the collection frequency was generally consistent across animals. In total, 3254 semen records were collected. During this year-long period, the boars were housed individually in crates 24–28 inches wide with partially slatted concrete floors and fed approximately 5–6 lbs of sow gestation feed once daily, either by hand or automatically. Occasionally, boars showing signs of lameness were treated according to standard farm health protocols and, if possible, moved to larger pens for improved mobility. These cases were sporadic and temporary and were therefore unlikely to systematically affect semen quality evaluation. They were kept in a tunnel-ventilated, negative-pressure barn, with temperatures maintained between 50 and 85 °F throughout the year. As all boars were housed in the same facility and evaluated repeatedly over time, environmental variation, including temperature, was shared across individuals and unlikely to act as a confounding factor in differentiating individuals.

The B-ultrasound imaging system used in this study was the ExaPad Mini (IMV imaging, L’Aigle, France) equipped with an LR 760 V Veterinary Rectal Linear Probe. The device was configured with standardized settings: a depth of 80 mm to capture the entire target area, a focus of 60 mm for optimal clarity, and a frequency of 10.0 MHz to balance resolution and penetration. Brightness-related parameters, including a gain of 65 and power at 50%, were maintained consistently to ensure uniform image quality and eliminate variability from manual adjustments. A farm employee trained in animal handling and ultrasonography performed ultrasonographic examinations of the boar testes while the boars remained standing in their original housing system, which consisted of individual stalls. Ultrasonography is a non-invasive and painless procedure that does not require additional animal restraint, handling, or the use of analgesics. The probe was applied gently to the testicle surface with conductive gel to enhance contact and eliminate air gaps, ensuring clear images. Two probe orientations were used to capture cross-sectional views: vertical, with the probe positioned perpendicularly to the testicle for longitudinal images, and horizontal, placed parallel to the surface for transverse views. Each boar was scanned to obtain 4 to 5 horizontal images and 8 to 10 vertical images, providing comprehensive visualization of the testicular structure. Fewer horizontal images were required because the transverse orientation allowed both testes to be visualized simultaneously, whereas vertical (longitudinal) scans were performed separately for each testis.

**Figure 2:**
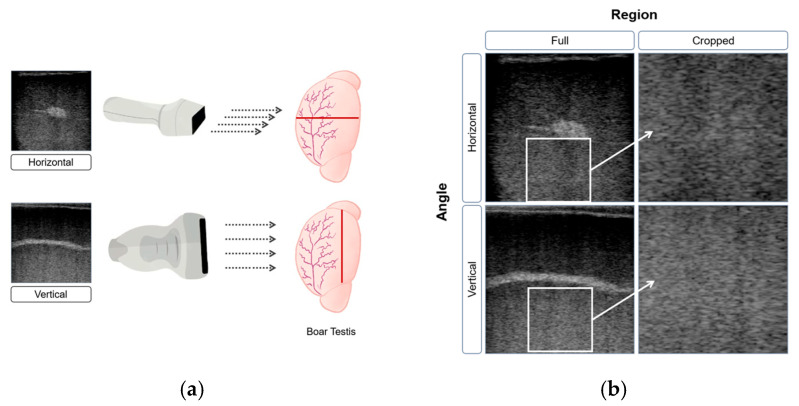
(**a**) B-ultrasound images of boar testes from horizontal and vertical orientations. The red line indicates probe contact orientation with the testis, and the gray arrows indicate scanning direction; (**b**) each image was processed in two formats: full images and cropped sections. The white boxes in the full images (**left**) indicate the regions extracted for the cropped versions (**right**).

Figure 2a illustrates the two probe orientations used to capture complementary cross-sectional views, each contributing unique anatomical details for analysis. These distinct perspectives were critical for feature extraction and model training. The rete testis, a dense central structure responsible for collecting sperm from the seminiferous tubules, appears as a bright white band in vertical view due to its high density and strong sound reflection, while in horizontal view it is visible as a small bright point. In contrast, the seminiferous tubules, coiled and occupying most of the testicular volume, are enclosed within the tunica albuginea and appear as shadowy regions in ultrasound images due to their lower density and weaker sound reflection.

Semen evaluation, assessing both quality and quantity, began when the boars were 6 to 9 months old. Each boar was collected from 2 to 49 times, with a median of 30 collections. A small number of boars were culled after only a few collections due to reasons such as semen quality, genetic assessments, or anatomical evaluations. A total of nine boars were culled due to poor semen quality after only a few collections. These individuals were retained in the dataset and were classified as underperforming according to the study criteria. Semen quality for each collection was evaluated based on sperm morphology and motility. Morphology was assessed by the percentage of sperm with intact structures, such as a complete head and tail, and no visible cytoplasmic droplets, observed under a phase-contrast brightfield microscope at 20× magnification. Motility was measured by the percentage of sperm displaying active movement. In addition to quality metrics, semen volume was also evaluated. Following each collection, an extender was added to the semen at an approximate 1:1 ratio to maintain viability. The recorded volume represents the total diluted amount, combining both semen and extender, to estimate the total volume per collection.

### 2.2. Target Variable and Data Preprocessing

This study consisted of two phases. The first phase aimed to predict the potential for high-quality semen production in boars using a DL model trained on early testicular B-ultrasound images. To effectively predict the potential for high-quality semen production in boars, it was first necessary to establish clear criteria for classifying boars as high-quality or underperforming based on their semen quality. Semen samples were collected at intervals of 5–14 days throughout 2022, allowing semen quality performance to be monitored across a significant developmental period. Semen quality was assessed according to industry standards, focusing on collections obtained from boars between approximately 200 days of age (first collection) and 18 months of age (~540 days), a range selected because total sperm count per collection typically reaches a plateau around 18 months before gradually declining. The dataset included records for 107 boars, with each boar having multiple semen samples collected at varying intervals over approximately one year. Invalid samples were discarded by the technician due to the low quality of the semen sample. The evaluation considered three semen quality metrics: total sperm count, motility, and morphology. A collection was classified as a good collection if it met all three criteria: total sperm count of ≥ 20 × 10^9^, motility > 70%, and morphology > 70% [11]. Collections meeting only one or none of these criteria were classified as bad collections. Additionally, some samples were retained as valid collections with corresponding measurements recorded. However, if the technician labeled these samples as having poor semen quality, we categorized them as trash collections, indicating that, despite having recorded values, these samples were considered unacceptable for further use.

**Figure 3:**
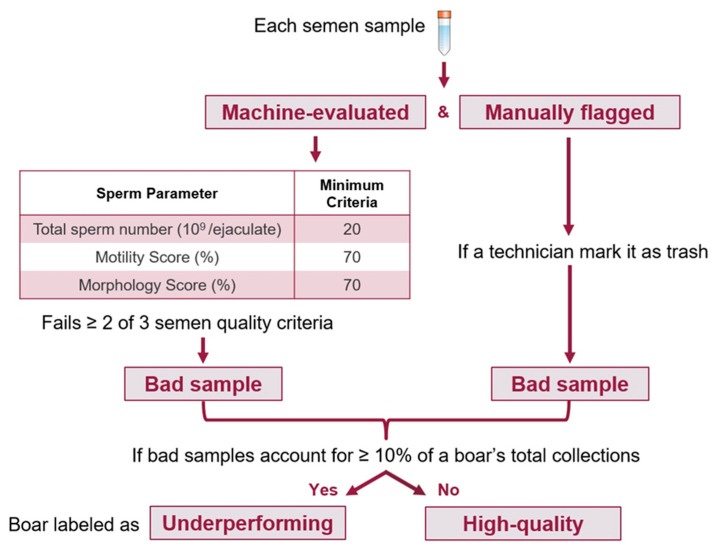
Semen quality-based classification of boars. Samples failing ≥ 2 criteria or manually flagged as trash were designated as bad. Boars with 10% or more bad samples were classified as underperforming, while the rest were considered high-quality.

As shown in Figure 3, a two-step process was employed to identify underperforming boars while reducing misclassification due to isolated poor collections. First, a boar was required to exhibit more than one low-quality collection (either bad or technician-flagged trash samples). Second, if the combined number of bad and trash collections accounted for at least 10% of the boar’s total collections, the boar was classified as underperforming. This 10% threshold reflects common operational tolerances in large-scale commercial swine production and was adopted as a pragmatic cutoff to balance biological relevance with statistical stability, acknowledging that occasional low-quality collections may arise from transient environmental or procedural factors rather than deficiencies in intrinsic reproductive potential.

**Figure 4:**
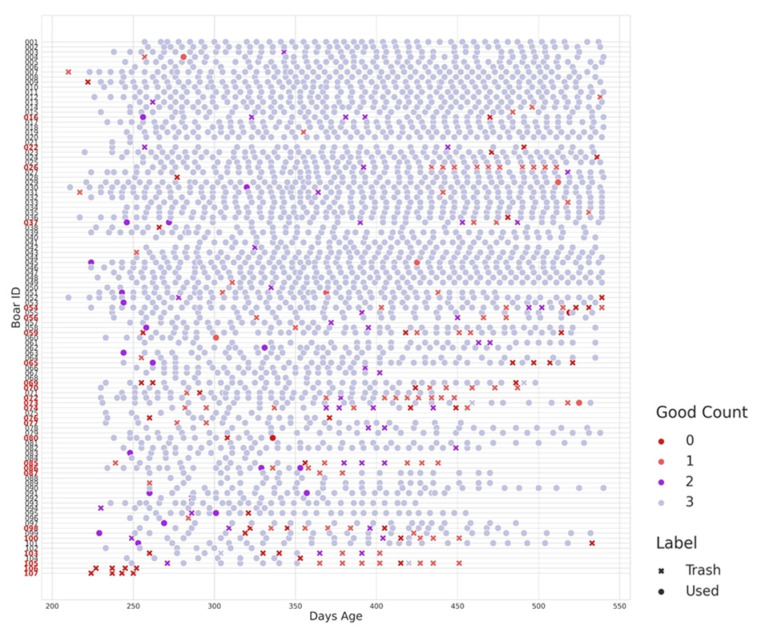
Good Count indicates the number of criteria met for a good collection: total sperm count ≥ 20 × 10^9^, motility > 70%, and morphology > 70%. The label indicates valid samples marked as trash collections due to poor semen quality. The 25 boars highlighted in bold red on the *x*-axis represent individuals classified as underperforming.

Applying this classification method, 82 boars were categorized as high-quality, consistently meeting the semen quality standards throughout the assessment period. In contrast, 25 boars were identified as underperforming based on the frequency and proportion of low-quality collections relative to their total collections. Additionally, as shown in Figure 4, underperforming boars exhibit frequent trash collections (red crosses) and low-quality collections (Good Count = 0, red dots) throughout the sampling period. These patterns are particularly evident between 250 and 300 days of age but persist in some boars even beyond 400 days. The lack of high-quality collections (Good Count = 3) further indicates consistent poor performance rather than isolated low-quality samples. Detailed individual-level semen evaluation data used for classification are provided in Supplementary Table S1, complementing the temporal patterns of semen quality illustrated in Figure 4. It is noted that these binary labels are solely for improving statistical power and modeling purposes. They do not directly reflect animal semen quality based on industry standards.

Building on the exploration of the prediction’s potential, the second phase of the study aimed to identify scanning factors that significantly influence the model’s predictive performance. To achieve this, the images underwent three different types of pre-processing and were divided into subgroups for further evaluation. The first factor considered was the region of interest, with images divided into original B-ultrasound views and cropped images focused on the central testicular region, specifically the seminiferous tubules, to highlight key areas relevant to semen production, as demonstrated in Figure 2b. The images were grouped into full and cropped sets for training. The study compared model performance across both sets to assess whether excluding bright, echoic regions affected the prediction of semen production capacity.

The second factor examined was the angle from which the ultrasound images were captured. Two angles were considered: horizontal view and vertical view. In the horizontal view, the ultrasound waves traveled across the horizontal cross-section of the testicle, capturing more information from the seminiferous tubules and vas deferens, which are key structures involved in sperm production and transport. In contrast, the vertical view showed the vertical cross-section, where the ultrasound waves interacted more with the rete testis, tunica albuginea and epididymis, crucial for sperm maturation and storage. These different anatomical perspectives could influence the model’s learning process. By comparing the predictive power of models trained on horizontal versus vertical view images, the study aimed to determine which angle provided greater accuracy in identifying boar semen production capacity.

**Figure 5:**
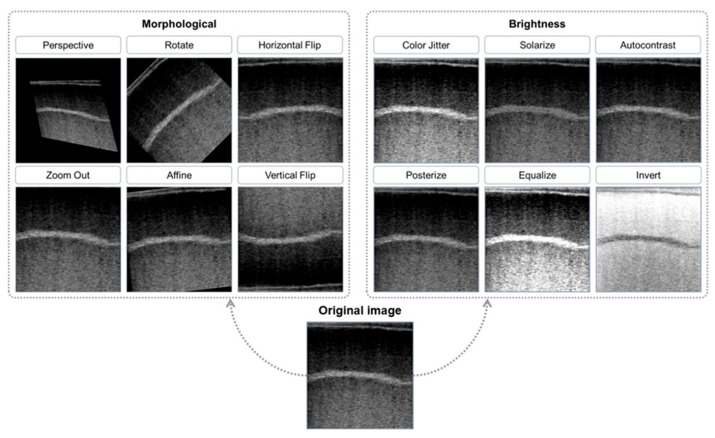
Image augmentation techniques applied to testicular ultrasound images, including morphological transformations (e.g., perspective, rotation, flipping) and brightness adjustments (e.g., color jitter, solarization, autocontrast). These augmentations were used to assess their impact on model performance in predicting semen quality.

The third factor was the data augmentation. Data augmentation is common in computer vision tasks, diversifying the image by applying random transformations or pixel value shifts to the image matrix. This study leverages the nature of the data augmentation that also randomly changes the image grayscale intensity patterns that may reflect underlying tissue echogenicity in B-ultrasound images, to investigate how the image brightness affects the model’s prediction power. A brief description and visual inspection of augmented images are shown in Table 1 and Figure 5, respectively. There are two types of augmentations: one focused on morphological transformations, including perspective, rotation, horizontal flip, zoom out, affine, and vertical flip; the other on brightness adjustments, including color jitter, solarize, autocontrast, posterize, equalize, and invert. The study included two augmentation strategies: the “Comprehensive Augmentation” group, where both brightness and morphological transformations were applied, and the “Morphological Augmentation” group, where only morphological transformations were used, preserving the original brightness. This setup aimed to assess whether brightness adjustments significantly influenced the model’s ability to predict semen quality. By comparing the two experimental groups, Morphological Augmentation and Comprehensive Augmentation, the study evaluated how removing brightness features affected overall model performance.

### 2.3. Model Selection and Evaluation

The YOLOv11 Model was adapted for image classification in this study, transitioning from its original design for object detection. Instead of detecting multiple objects, the model classifies an entire image into a single label (e.g., high-quality or underperforming boar) [15]. Two versions of YOLOv11 were tested: YOLOv11n (nano) and YOLOv11m (medium). YOLOv11n is a lighter, faster variant with fewer parameters and a lower FLOP count, making it more efficient for real-time applications and resource-constrained environments. Both models were tested to balance speed and accuracy, with YOLOv11n preferred for speed and YOLOv11m used to evaluate if its higher accuracy could better capture the subtle details needed for semen quality classification. Transfer learning further improves YOLOv11’s performance, making it suitable for this task despite limited data [18].

Models were trained for 100 epochs using the SGD optimizer (momentum = 0.937, weight decay = 0.0005) and a cosine learning rate schedule. To implement the described experimental groups, standard YOLO color augmentations were disabled. Morphological transformations were applied as the baseline, while brightness-altering operations were strictly reserved for the “Comprehensive Augmentation” group to isolate grayscale intensity effects.

**Figure 6:**
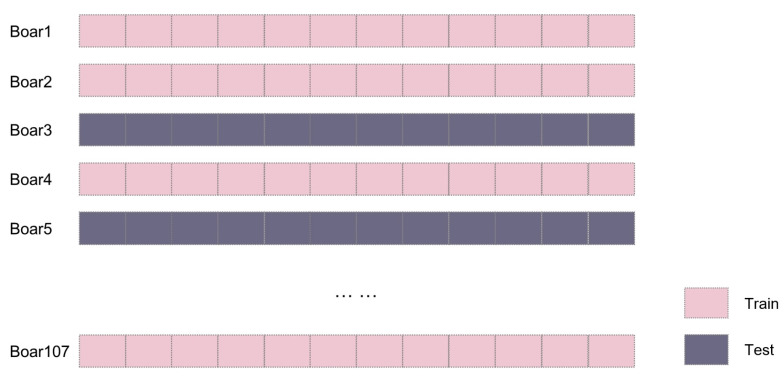
Images from the same boar are placed in either the train or test set using an 80/20 split. Each rectangle represents one ultrasound image from a boar. Each boar has 9–17 images, simplified here to 12 (the median) for clarity.

The dataset was partitioned using a By-ID Split strategy to maintain strict subject-level independence, as illustrated in Figure 6. Unique animal identifiers were randomly assigned to training, validation, or test sets via a 5-fold cross-validation framework. This approach groups all ultrasonic images of a specific boar into a single subset, forcing the YOLOv11 model to learn generalizable quality features rather than recognizing individual anatomical markers. By isolating the model from test subjects during training, the framework provides a robust assessment of its performance on unseen individuals.

Final performance was evaluated by aggregating image predictions for each boar using the SemenEval class. An Aggregated Inference strategy was applied by averaging the probability scores of all test images associated with a specific boar ID. This mean probability was compared against an optimized threshold of 0.3 for final classification. This decision-level calibration maintains the natural population prevalence while ensuring that metrics reflect the accuracy in categorizing animals rather than isolated records. This methodology aligns the results with practical breeding decisions, where a single conclusion is required for each animal despite variability across independent observations. High-quality boars were defined as the positive class, while underperforming boars were treated as the negative class. Model performance was quantified using the following metrics:

- Precision: This metric represents the proportion of correctly identified high semen-potential boars among those classified as high semen-potential. High precision reduces false positives, ensuring that only boars with strong reproductive potential are selected.
- Recall: This metric indicates the proportion of actual high semen-potential boars successfully identified by the model. High recall ensures comprehensive identification, reducing the likelihood of overlooking boars with optimal breeding qualities.
- F1-score: As the harmonic mean of Precision and Recall, the F1-score provides a balanced evaluation of the model’s overall diagnostic performance. It is calculated as follows:

$$F_{1}=2\times \frac{Precision\times Recall}{Precision+Recall}$$

### 2.4. Statistical Design and Experimental Evaluation Framework

Multiple independent trials were conducted across 16 experimental configurations using subject-level partitioning. Across all runs, a total of *n* = 869 configuration–run evaluations were available for statistical analysis, with most runs containing all 16 configurations and a small number containing partial outputs due to incomplete runs. Each configuration–run combination was treated as an independent observation, while animal-level independence was preserved through By-ID splitting to prevent information leakage across training and test sets. Table 2 summarizes the four primary study factors, which were evaluated for their individual and combined influence on model performance.

The statistical evaluation was conducted using a General Linear Model (GLM) to quantify the independent contribution of each experimental factor to the model’s performance. Within this unified GLM framework, analysis of variance (ANOVA) was used to assess the statistical significance of main effects, while ordinary least squares (OLS) estimation was employed to quantify the direction and magnitude of each factor’s contribution. The main effects model followed the formula:

$$y_{ijkm}=\;\mu +\;R_{i}+\;A_{j}+\;M_{k}+\;B_{m}+\;\epsilon_{ijkm}$$

Here, $y_{ijkm}$ represents the observed performance for a specific combination of factors. The intercept μ represents the overall mean performance, and $\epsilon_{ijkm}$ is the random error term. The indices *i*, *j*, *k*, m correspond to the levels of the factors: $R_{i}$ represents the region of the ultrasound images (Full image or Cropped section), $A_{j}$ denotes the angle at which the ultrasound was captured (Horizontal view or Vertical view), $M_{k}$ refers to the DL model used (YOLOv11n or YOLOv11m), and $B_{m}$ corresponds to whether brightness augmentation was applied (Comprehensive Augmentation or Morphologic al Augmentation).

Building upon the main effects analysis, a Two-Way Interaction ANOVA investigated whether the impact of one scanning or processing factor depended on the levels of another. The statistical model was expanded to include all first-order interaction terms:

$$y_{ijkm}=\;\mu +\;R_{i}+\;A_{j}+\;M_{k}+\;B_{m}+{\left(R\;\times A\right)}_{ij}+{\left(R\;\times M\right)}_{ik}+{\left(R\;\times B\right)}_{im}+{\left(A\;\times M\right)}_{jk}+{\left(A\;\times B\right)}_{jm}+{\left(M\;\times B\right)}_{km}+\epsilon_{ijkm}$$

In this expanded model, the terms ${\left(R\;\times \;A\right)}_{ij}$, ${\left(R\;\times \;M\right)}_{ik}$, ${\left(R\;\times \;B\right)}_{im}$, ${\left(A\;\times \;M\right)}_{jk}$, ${\left(A\;\times \;B\right)}_{jm}$, ${\left(M\;\times \;B\right)}_{km}$ represent the two-way interaction effects between the primary factors. These terms account for dependencies where the influence of one factor on performance is contingent upon the level of another factor rather than being strictly additive. The paired indices denote the specific combinations of factor levels being evaluated. Significant synergies or antagonisms were identified via F-tests, with statistical significance established at *p* < 0.05. All statistical analyses were implemented using the Statsmodels library (version 0.14.0) in Python (version 3.11).

Model assumptions were verified through diagnostic tests. No evidence of strong residual autocorrelation was observed (Durbin–Watson = 1.869), and the Condition Number of 4.23 indicated minimal multicollinearity. The Durbin–Watson statistic was used to assess first-order autocorrelation. Because the data were not time-ordered, higher-order autocorrelation was not evaluated. Although residuals deviated from normality, the large sample size (*n* = 869 configuration–run evaluations) supports the robustness and validity of the ANOVA and OLS results.

## 3. Results

Table 3 summarizes the mean and standard deviation of performance metrics across 16 experimental configurations. The highest precision (88.20%) was achieved using cropped images with a vertical scanning angle and morphological augmentation on the YOLOv11n model. However, the lack of variability (±0.00) in this configuration indicates no variation across runs. In contrast, the configuration utilizing full images, a vertical scanning angle, and morphological augmentation with YOLOv11m achieved the highest recall (99.33 ± 2.57%) and F1-score (91.33 ± 2.20%). This balanced performance indicates strong classification capability and effectively minimizes the risk of misidentifying high-quality boars. Notably, configurations combining full images with vertical scanning consistently produced recall values above 91.33%.

**Figure 7:**
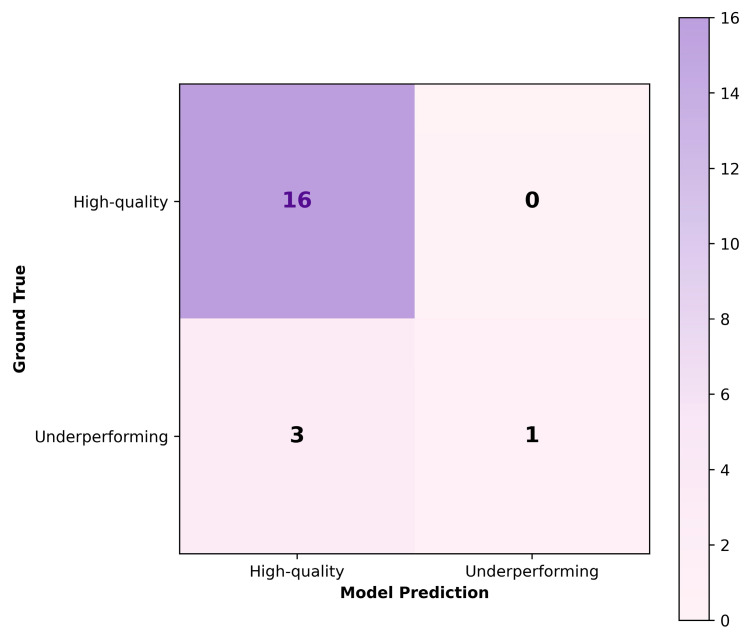
Confusion matrix for an independent test set (20 boars) evaluated using the best-performing configuration, complementing the aggregate results summarized in Table 3.

To provide a decision-level illustration of this configuration, Figure 7 presents the confusion matrix obtained on an independent test set of 20 boars, showing precision, recall, and F1-score values consistent with the aggregate statistics reported in Table 3.

### 3.1. Main Effects Analysis

The main effects of each experimental factor on classification performance were evaluated using ANOVA, with corresponding results reported in Table 4, and further quantified using OLS regression in Table 5.

As shown in Table 4, Region exerted the strongest influence on recall, with a sum of squares of 9.1324 and an F statistic of 322.457 (*p*-value < 0.001), exceeding the effects of other factors. Consistent with this finding, OLS results in Table 5 indicate a large negative coefficient for cropped regions (−0.2049), indicating a substantial reduction in recall associated with cropping. While Region also significantly affected precision in Table 4, its effect size was comparatively modest, as reflected by a small positive coefficient (0.0118) in Table 5.

Scanning Angle was another determinant of recall performance. ANOVA results in Table 4 show a highly significant effect (F = 129.658, *p*-value < 0.001), and OLS estimates in Table 5 indicate that vertical scanning angles are associated with higher recall (coefficient = 0.1302). This increase in recall was accompanied by a slight but statistically significant reduction in precision, as indicated by a negative coefficient for vertical angles (−0.0144) in Table 5.

Augmentation strategy had the most pronounced impact on precision. As reported in Table 4, augmentation exhibited the largest F statistic for precision (F = 150.281, *p*-value < 0.001). OLS results in Table 5 show that comprehensive augmentation, including brightness adjustments, is associated with reduced precision (coefficient = −0.0334). Augmentation had a statistically significant but relatively small positive effect on recall in Table 5 (coefficient = 0.0376).

Model architecture had a negligible influence on recall, as indicated by a non-significant F statistic in Table 4 (F = 0.361, *p*-value = 0.5481) and a near-zero OLS coefficient in Table 5. Although YOLOv11n showed a statistically significant difference in precision relative to YOLOv11m in Table 5, the magnitude of this effect was the smallest among all significant factors.

### 3.2. Factor Distributions and Interactions

**Figure 8:**
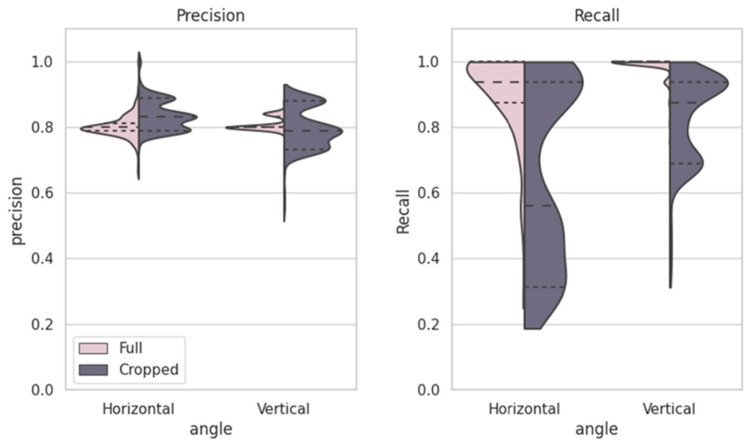
The violin plots show recall and precision distributions for different image angles (horizontal and vertical) and regions (full and cropped). Horizontal images generally achieve higher recall, especially for full images, while cropped images consistently show better precision with more compact distributions.

Figure 8 illustrates the distributions of precision and recall across scanning angles (horizontal vs. vertical) and image regions (full vs. cropped). Full images exhibit higher recall values, particularly under vertical scanning, whereas cropped images show more compact precision distributions with lower recall.

The ANOVA results in Table 4 confirm that image region, scanning angle, and augmentation strategy each exerted a statistically significant effect on all three performance metrics (*p*-value < 0.001). Notably, model architecture influenced precision and F1-score but did not have a statistically significant effect on recall (F = 0.361, *p*-value = 0.548). Furthermore, Table 6 shows significant two-way interactions, such as Angle × Augmentation (F = 189.0419 for recall). The significant Region × Augmentation interaction across all metrics is also observed across configurations. These interaction effects are consistent with the performance variability observed across the configurations summarized in Table 3.

## 4. Discussion

The dataset of 1417 ultrasound images from 107 boars presented challenges in achieving model generalization, as a larger dataset would likely capture a broader range of physiological variations. To enhance model performance under these limitations, transfer learning with YOLOv11 was applied, using pre-trained weights to detect complex anatomical features without requiring manual feature labeling. YOLOv11 is effective at identifying subtle patterns, which is valuable in ultrasound analysis, where reproductive health indicators appear as slight differences in tissue structure, density, and texture [19]. Data augmentation techniques, including rotations, scaling, and flips, were used to increase dataset variability and improve model robustness [20,21]. Consistency in image acquisition was also prioritized; unlike in clinical settings, where operators adjust brightness and contrast based on the acoustic window, all images in this study were captured under uniform settings, minimizing operator-induced variability [22,23]. The observed reduction in precision following brightness augmentation suggests that pixel intensity, as a representation of echogenicity, plays a biologically meaningful role in model prediction. In testicular ultrasound, echogenicity, reflected as pixel brightness, is directly associated with cellular density, fluid content, and the proportion of connective tissue, all of which are critical for spermatogenesis. As a result, artificial changes in image brightness during augmentation may disrupt these biologically relevant intensity patterns, contributing to the observed reduction in model precision. This indicates that the model is capturing biologically meaningful variations in testicular tissue composition that are directly associated with spermatogenic function and subsequent semen quality.

In reproductive ultrasound analysis, established methods such as ImageJ and Ecotext quantify testicular echotexture through predefined biological features, including pixel intensity distributions and microstructural parameters such as seminiferous tubule density and lumen area [12,13]. These approaches provide direct biological interpretation but rely on manual or semi-structured feature extraction. In contrast, the current study applies a deep learning model that learns from raw images without explicitly defining these features. The limited difference observed between YOLOv11n and YOLOv11m suggests that increasing model capacity alone does not substantially improve performance, indicating that the primary constraint lies in the input representation rather than computational complexity. This further supports that feature representation, rather than model capacity, is the primary limitation. This interpretation is consistent with the augmentation results, where brightness adjustments significantly reduced precision, suggesting that grayscale intensity, which reflects tissue echogenicity, is a key feature used by the model. Similarly, the improved performance of full images compared to cropped regions indicates that the model utilizes broader anatomical context, including structures beyond the seminiferous tubules, to support prediction. These findings suggest that the model captures biologically relevant information, rather than relying on random image noise or irrelevant artifacts, although this is achieved implicitly rather than through structured feature extraction. As a result, the lack of explicit biological descriptors may limit interpretability and constrain further performance improvement. Nevertheless, the consistent performance across configurations suggests that the model is implicitly learning biologically relevant tissue characteristics rather than relying on spurious image patterns.

In this study, region, angle, and augmentation significantly influenced model performance, with region and augmentation having the most substantial impact. Considering the research objective of accurately identifying high-quality boars to minimize costly misclassifications, precision is prioritized over recall. Augmentation, particularly random brightness adjustments, had the most detrimental effect on precision (*p*-value < 0.001, coefficient = −0.0334), highlighting that preserving original brightness is crucial since pixel brightness reflects tissue density [24]. At the same time, augmentation exhibited a modest but statistically significant positive effect on recall. This trade-off suggests that brightness augmentation may increase model sensitivity at the expense of specificity, leading to more aggressive positive predictions. In comparison, region had a smaller but positive effect on precision (*p*-value < 0.001, coefficient = 0.0118). The findings also indicate that using full B-ultrasound images consistently improves precision, as they retain essential anatomical details such as the rete testis, tunica albuginea, and epididymis, which are often lost when images are cropped. Although seminiferous tubules are directly responsible for sperm production, excluding these surrounding anatomical landmarks may remove critical contextual cues reflecting overall testicular development and maturity, which are particularly important for early-life prediction. This additional structural context reduces false positives by providing a more comprehensive representation of reproductive tissue integrity [25]. Regarding imaging angles, vertical angles slightly improve recall (*p*-value < 0.001, coefficient = 0.1302) by capturing more relevant details from the rete testis structure. However, this enhancement comes at the expense of a minor reduction in precision (*p*-value < 0.001, coefficient = −0.0144), suggesting a trade-off between recall and precision. Nevertheless, the negative impact of vertical angles on precision is far less significant compared to the substantial influence of augmentation, making it a minor concern in optimizing the model’s performance. Moreover, horizontal views generally provide stable texture and density details, which further supports precision by enhancing the model’s ability to detect subtle variations in tissue health and composition [26]. These findings emphasize that prioritizing precision requires using full images, preserving brightness information, and carefully balancing angle selection to enhance model reliability.

The high recall achieved by YOLOv11n in vertical-angle full images suggests a state of high numerical stability where the model produced consistent results across trials. This performance likely indicates that the limited architectural capacity of the model, which features 1.5 M parameters and 6.5 B FLOPs, leads to rapid convergence on specific features within the training data. While this result produces high recall for the current dataset, it may suggest a trade-off in the model’s ability to generalize to new or unseen data environments. In contrast, YOLOv11m, with its more complex architecture and larger number of parameters, exhibited a better balance between accuracy and recall. While YOLOv11m achieved a recall of 99.33 ± 2.57% in the full image, vertical angle, and morphological augmentation configuration, its performance showed greater stability, indicating that the model was able to learn from broader patterns without excessive overfitting. The difference in performance between YOLOv11n and YOLOv11m highlights the trade-off between model size and generalizability. YOLOv11n, with fewer parameters (1.5 M) and lower computational demands (6.5 B FLOPs), is more computationally efficient, processing images faster (1.5 ms), but tends to overfit due to its smaller capacity. In contrast, YOLOv11m, with more parameters (20.1 M) and higher computational demands (68.0 B FLOPs), is slower (4.7 ms) but offers better accuracy and stability, leveraging its larger capacity to generalize more effectively and capture more robust features. Thus, while YOLOv11n is ideal for real-time performance, YOLOv11m provides higher accuracy and stability at the cost of processing speed [18].

The By-ID Split method, which groups all images from each pig within a single subset, offers a more realistic evaluation of model performance in real-world scenarios where new, unseen individuals are encountered. Unlike By-Image Split, which randomly divides individual images across training and test sets, By-ID Split forces the model to learn broader patterns related to semen quality rather than relying on individual-specific features. This approach enhances the model’s generalizability and robustness when ap-plied to new animals [27]. While By-Image Split, which divides images randomly without considering individual identities, may achieve higher internal accuracy due to repeated exposure to the same individuals, it risks overfitting and provides an overly optimistic assessment of model performance [28]. In contrast, By-ID Split prioritizes generalization, which is crucial for developing a model that can reliably predict semen quality across diverse populations. Although this method may slightly reduce internal accuracy by challenging the model to learn from more generalizable features, it ensures better performance on unseen data, making it more suitable for real-world applications [29].

As shown in Figure 4, high semen-potential boars constitute the majority of the population, while underperforming boars are relatively few and exhibit diverse characteristics without a clear classification threshold. This distribution highlights the practicality of defining high semen-potential boars as the “positive” category, particularly since our objective is to identify patterns associated with superior reproductive performance. Prioritizing the identification of high semen-potential boars enables the model to focus on detecting traits linked to optimal semen quality, thereby enhancing precision in selection processes. Early identification of these boars allows for strategic allocation of breeding resources, which aligns with the goal of minimizing the risk of falsely classifying poor-performing boars as high-quality ones. Concentrating the model’s learning on high semen-potential cases improves its ability to capture traits associated with strong semen production, enhancing overall selection efficiency [30]. Conversely, defining low semen-potential boars as the positive category would bias the model toward detecting rare or inconsistent traits, potentially overlooking essential indicators of high semen quality [31].

Despite these findings, several limitations should be acknowledged. First, the relatively limited dataset size and the use of data from a single commercial farm may restrict the generalizability of the model to other populations and management systems. Second, although the model demonstrates reasonable predictive performance, the lack of explicitly defined biological features limits interpretability compared to traditional echotexture analysis methods. Finally, environmental and management factors, such as seasonal variation and housing conditions, were not explicitly modeled and may influence semen quality outcomes. Future research could focus on refining the predictive accuracy of ultrasound imaging for reproductive health by examining image brightness as a factor associated with high semen-potential boars. This study has demonstrated that using full images, rather than focusing solely on the seminiferous tubules, provides a more positive impact on model performance. This suggests that structures beyond the seminiferous tubules, such as the tunica albuginea and epididymis, may also contribute valuable information for prediction. Therefore, future studies should further investigate the roles of these anatomical structures in enhancing model accuracy, while also exploring the potential of Doppler ultrasound to measure blood flow within the testes [32]. Since blood flow relates directly to the seminiferous tubules, which are critical for spermatogenesis, Doppler ultrasound could provide valuable numerical data that would enhance feature extraction for DL models and improve high semen quality potential prediction [33].

In the longer term, developing a unified data platform across the livestock industry could support robust model training and generalization by standardizing ultrasound imaging protocols and equipment across different farms and regions. Such standardization would address the variability introduced by different equipment and operator settings, which can result in inconsistent measurements and hinder model consistency [34,35]. Additionally, expanding datasets to cover a wider range of physiological variations and rare conditions, along with fine-tuning models specifically for medical imaging, would address common data gaps in ultrasound studies [36]. Efforts to design models capable of addressing artifacts and noise could further improve prediction accuracy, ensuring that models are better suited to assess reproductive health across varied environments and imaging conditions.

## 5. Conclusions

This study aimed to predict semen production potential using early B-ultrasound images of boars’ testicles. The models extracted image features, focusing on anatomical structures such as seminiferous tubules, to assess their predictive value. Although the analysis revealed some relationship between testicular structures and boar potential for semen production, the overall accuracy of the models was not sufficient to reliably predict semen quality potential, indicating that these methods cannot yet replace traditional assessment approaches.

An analysis of factors, including region, angle, augmentation, and model, identified region, angle, and augmentation as the most influential factors on model performance. Specifically, the physiological structures beyond the shadowy regions of the testicular images, along with the brightness that reflects the intensity of ultrasound echoes, played a crucial role in the model’s predictive power. Preserving brightness features during augmentation was particularly important for distinguishing between high- and low semen-potential boars. These findings emphasize the importance of capturing full anatomical details, maintaining image quality, and applying thoughtful data processing techniques to enhance model predictions.

For future research, Doppler ultrasound could provide valuable insights into testicular function and semen quality by offering quantitative data on blood flow, closely linked to spermatogenesis. Expanding datasets across multiple farms and establishing a unified data platform in the livestock industry would support large-scale analysis, improve model generalizability, and strengthen predictive accuracy by capturing a broader range of physiological variations. Standardizing ultrasound protocols would also help reduce variability, enhancing model robustness in assessing reproductive health.

## Figures and Tables

**Table 1:** Comparison of Morphological Transformations and Brightness Adjustments.

Morphological Transformations	Brightness Adjustments
Perspective transformation	Color jitter
Rotation	Random solarization
Horizontal flip	Random autocontrast
Vertical flip	Random posterization
Random zoom out	Random equalization
Random affine transformation	Random inversion

**Table 2:** Summary of the Study Factors.

Factor	Description	Factor Level 0	Factor Level 1
Region	Comparison between full and cropped images	Full Image	Cropped Section
Angle	Comparison between horizontal view and vertical view images	Horizontal View	Vertical View
Aug. ^1^	Whether brightness augmentation was applied or not	Morphological Aug.	Comprehensive Aug.
Model	Comparison between YOLOv11m and YOLOv11n	YOLOv11m	YOLOv11n

^1^ “Aug.” is an abbreviation for “Augmentation”.

**Table 3:** Summary of Precision and Recall for Different Configurations.

Factor 1 (Region)	Factor 2 (Angle)	Factor 3 (Augmentation)	Factor 4 (Model)	Precision (%) ^1^	Recall (%) ^1^	F1-Score (%) ^1^
Full	Horizontal	Morphological Aug. ^2^	YOLOv11m	79.91 ± 2.50	94.43 ± 9.51	86.22 ± 5.25
			YOLOv11n	80.28 ± 2.48	93.98 ± 8.93	86.46 ± 4.86
		Comprehensive Aug.	YOLOv11m	80.20 ± 5.12	81.59 ± 19.54	80.90 ± 13.07
			YOLOv11n	80.18 ± 3.53	91.19 ± 12.61	85.06 ± 9.87
	Vertical	Morphological Aug.	YOLOv11m	84.04 ± 1.04	**99.33 ± 2.57 ^b^**	**91.33 ± 2.20 ^c^**
			YOLOv11n	80.00 ± 0.00	100.00 ± 0.00	88.89 ± 0.00
		Comprehensive Aug.	YOLOv11m	79.92 ± 1.05	97.24 ± 4.80	88.55 ± 3.53
			YOLOv11n	80.00 ± 0.00	100.00 ± 0.00	88.89 ± 0.00
Cropped	Horizontal	Morphological Aug.	YOLOv11m	87.83 ± 2.59	57.29 ± 16.68	69.34 ± 15.66
			YOLOv11n	83.30 ± 0.00	31.20 ± 0.00	45.43 ± 0.00
		Comprehensive Aug.	YOLOv11m	84.50 ± 5.48	80.22 ± 20.24	82.20 ± 16.51
			YOLOv11n	78.90 ± 0.00	93.80 ± 0.00	85.68 ± 0.00
	Vertical	Morphological Aug.	YOLOv11m	79.13 ± 2.24	90.95 ± 9.48	84.76 ± 6.40
			YOLOv11n	**88.20 ± 0.00 ^a^**	93.80 ± 0.00	90.92 ± 0.00
		Comprehensive Aug.	YOLOv11m	78.52 ± 5.21	77.88 ± 15.58	78.20 ± 12.93
			YOLOv11n	73.30 ± 0.00	68.80 ± 0.00	70.93 ± 0.00

^1^ The Precision, Recall and F1-score values are presented as mean ± standard deviation (SD) for each configuration, with the highest performance bolded. ^2^ “Aug.” is an abbreviation for “Augmentation”, used consistently throughout the table. ^a^ The bolded Precision value (88.20 ± 0.00) represents the highest precision observed across all configurations. ^b^ The bolded Recall value (99.33 ± 2.57) represents the highest recall observed across all configurations. ^c^ The bolded F1 value (91.33 ± 2.20) represents the highest recall observed across all configurations.

**Table 4:** ANOVA Results for Precision.

	Factor	Sum Sq. ^1^	F Statistics ^2^	*p*-Value ^3^
Precision	Region	0.0303	18.856	**<0.001 ^a^**
	Angle	0.0439	27.301	**<0.001**
	Aug. ^4^	0.2418	150.281	**<0.001**
	Model	0.0333	20.707	**<0.001**
	Residual ^5^	1.3903	-	-
Recall	Region	9.1324	322.457	**<0.001**
	Angle	3.6721	129.658	**<0.001**
	Aug.	0.3069	10.835	**0.001**
	Model	0.0102	0.361	0.5481
	Residual	24.4696	-	-
F1-score	Region	2.7896	240.051	**<0.001**
	Angle	1.3769	118.489	**<0.001**
	Aug.	0.0707	6.080	**0.0139**
	Model	0.0484	4.165	**0.0416**
	Residual	10.0403	-	-

^1^ Sum Sq. (Sum of Squares) represents the total variation attributed to each factor in the model. ^2^ F statistics is the ratio of the variance explained by a factor to the unexplained variance, used to test the significance of that factor. ^3^ *p*-value indicates the probability that the observed effect is due to random chance, with values less than 0.001 indicating strong significance. ^4^ “Aug.” is an abbreviation for “Augmentation”. ^5^ Residual refers to the unexplained variation in the data after accounting for the factors in the model. ^a^ Bolded values indicate statistical significance at *p*-value < 0.05.

**Table 5:** OLS Regression Results for Precision.

	Factor Level	Coefficient ^1^	*t* Statistics ^2^	*p*-Value ^3^	C.I. (95%) ^4^
Precision	Region (Cropped)	0.0118	4.353	**<0.001 ^a^**	[0.007, 0.017]
	Angle (Vertical)	−0.0144	−5.302	**<0.001**	[−0.020, −0.009]
	Aug. ^5^ (with Aug.)	−0.0334	−12.254	**<0.001**	[−0.039, −0.028]
	Model (YOLOv11n)	−0.0124	−4.551	**<0.001**	[−0.018, −0.007]
	Const. ^6^	0.8356	-	-	-
Recall	Region (Cropped)	−0.2049	−17.944	**<0.001**	[−0.227, −0.183]
	Angle (Vertical)	0.1302	11.405	**<0.001**	[0.108, 0.153]
	Aug. (with Aug.)	0.0376	3.292	**0.001**	[0.015, 0.060]
	Model (YOLOv11n)	−0.0069	−0.601	0.548	[−0.029, 0.016]
	Const.	0.8671	-	-	-
F1-score	Region (Cropped)	−0.1132	−15.479	**<0.001**	[−0.128, −0.099]
	Angle (Vertical)	0.0797	10.897	**<0.001**	[0.065, 0.094]
	Aug. (with Aug.)	0.0181	2.468	**0.014**	[0.004, 0.032]
	Model (YOLOv11n)	−0.0149	−2.041	**0.042**	[−0.029, −0.001]
	Const.	0.8258	-	-	-

^1^ Coefficient represents the estimated effect of a factor on the dependent variable. ^2^ *t* statistics is the test statistic used to determine the significance of each coefficient, calculated as the coefficient divided by its standard error. ^3^ *p*-value indicates the probability that the observed effect is due to random chance, with values less than 0.001 indicating strong statistical significance. ^4^ C.I. (95%) represents the 95% confidence interval for the coefficient, showing the range within which the true value is likely to fall. ^5^ “Aug.” is an abbreviation for “Augmentation”. ^6^ Const. (Constant) represents the baseline value of the dependent variable when all other factors are zero. ^a^ Bolded values indicate statistical significance at *p*-value < 0.05.

**Table 6:** Comprehensive ANOVA (F-statistics) for All Two-Way Interactions.

Factor	df ^1^	F Statistics ^2^		
		Precision	Recall	F1-Score
Main Effects				
Region	1	25.5146	439.8106	333.3599
Angle	1	37.7002	177.7388	165.3286
Aug. ^3^	1	202.5702	14.7655	8.4194
Model	1	27.8702	0.4659	5.7116
Interaction				
Region × Angle	1	100.5281	19.3583	26.2465
Region × Aug.	1	105.1253	73.3937	60.8956
Region × Model	1	2.0761	16.3044	19.1045
Angle × Aug.	1	41.5720	189.0419	213.2169
Angle × Model	1	24.7482	0.0104	4.5042
Aug. × Model	1	35.0769	26.5630	23.1042
Residual ^4^	858.0	-	-	-

^1^ df (Degrees of Freedom) represents the number of independent observations used to calculate a statistic, determined by the number of levels in each factor. ^2^ F statistics is the ratio of the variance explained by a factor to the unexplained variance, used to test the significance of that factor. ^3^ “Aug.” is an abbreviation for “Augmentation”. ^4^ Residual refers to the unexplained variation in the data after accounting for the factors in the model.

## Data Availability

The data presented in this study are available on request from the corresponding author. The data are not publicly available due to commercial confidentiality and company privacy policies related to production performance.

## Supplementary Materials

The following supporting information can be downloaded at: https://www.mdpi.com/article/10.3390/ani16081131/s1, Table S1: Individual-Level Semen Quality Summary and Classification of Boars.

## Abbreviations

The following abbreviations are used in this manuscript:

AI	Artificial intelligence
ANOVA	Analysis of variance
CT	Computed tomography
CV	Computer vision
DL	Deep learning
ML	Machine learning
RMI	Magnetic resonance imaging
PLF	Precision livestock farming
PQA+	Pork quality assurance plus
Sum Sq.	Sum of squares
