# Supplementary material for: Evaluating the Impact of Scanning Factors on Ultrasound Imaging for Predicting Semen Quality in Boars
Source: Animals (Basel). 2026 Apr 8;16(8):1131. doi: 10.3390/ani16081131 (PMC13113066; doi:10.3390/ani16081131)
Supplement: Supplementary file 1 [file animals-16-01131-s001.zip › animals-4161044-supplementary.pdf]

Table S1. Individual-Level Semen Quality Summary and Classification of Boars.

ID[1]	Birthdate	Pass Sperm Number[2] (%)	Pass Motility[3] (%)	Pass Morphology[4] (%)	Discard Rate[5] (%)	Culling[6]	Label
1	5/1/2021	100	100	100	0	Retained	High-quality
2	4/29/2021	97.4	93.4	93.4	5.3	Retained	High-quality
3	5/20/2021	100	100	98.4	1.6	Retained	High-quality
4	5/22/2021	100	96.4	96.4	1.8	Retained	High-quality
5	5/6/2021	100	100	98.6	0	Retained	High-quality
6	5/13/2021	100	100	100	0	Retained	High-quality
7	6/10/2021	98.9	96.6	85.4	6.9	Retained	High-quality
8	5/20/2021	100	100	100	0	Retained	High-quality
9	6/3/2021	98.9	97.7	95.5	4.5	Retained	High-quality
10	5/19/2021	100	97.8	97.8	2.2	Retained	High-quality
11	5/6/2021	100	100	100	0	Retained	High-quality
12	5/28/2021	100	98.9	93.1	6.9	Retained	High-quality
13	5/6/2021	98.1	100	100	1.9	Retained	High-quality
14	5/6/2021	98.8	97.5	97.5	3.8	Retained	High-quality
15	6/3/2021	100	98.4	98.4	1.6	Retained	High-quality
16	5/8/2021	96.7	93.4	98.4	16.7	Retained	Underperforming
17	5/26/2021	100	100	100	0	Retained	High-quality
18	5/4/2021	98.4	100	98.4	3.1	Retained	High-quality
19	5/18/2021	100	97.1	97.1	2.9	Retained	High-quality
20	5/6/2021	100	98	98	2	Retained	High-quality
21	5/27/2021	100	100	100	0	Retained	High-quality
22	5/27/2021	94.2	96.2	98.1	16.7	Culled	Underperforming
23	6/9/2021	98.8	98.8	98.8	1.2	Retained	High-quality
24	5/8/2021	97.7	98.8	96.5	4.7	Retained	High-quality
25	5/8/2021	98.2	98.2	98.2	1.8	Retained	High-quality
26	5/6/2021	100	73.7	71.1	28.9	Culled	Underperforming
27	6/3/2021	98.9	100	92.1	9	Retained	High-quality
28	5/13/2021	96.8	98.4	96.8	6.3	Retained	High-quality
29	5/30/2021	98.9	96.7	93.5	4.3	Retained	High-quality
30	6/9/2021	100	98.9	88.8	9.1	Retained	High-quality
31	6/3/2021	98.1	92.5	90.6	9.4	Retained	High-quality
32	5/27/2021	100	100	100	0	Retained	High-quality
33	4/29/2021	95.6	86.7	86.7	9.3	Retained	High-quality
34	5/27/2021	98.8	100	100	1.2	Retained	High-quality
35	5/6/2021	100	98.9	94.4	5.6	Retained	High-quality
36	6/3/2021	79.3	95.4	93.1	8.8	Retained	High-quality
37	5/18/2021	93.8	93.8	84.4	15.6	Retained	Underperforming
38	5/27/2021	98.1	98.1	98.1	1.9	Retained	High-quality
39	6/9/2021	100	100	100	0	Retained	High-quality
40	5/13/2021	100	100	100	0	Retained	High-quality
41	3/12/2021	100	100	100	0	Retained	High-quality
42	5/6/2021	100	100	98.1	1.9	Retained	High-quality
43	5/4/2021	98.8	98.8	97.7	2.3	Retained	High-quality
44	5/6/2021	100	96.5	84.7	7.6	Retained	High-quality
45	5/27/2021	98.9	98.9	83.3	5.6	Retained	High-quality
46	6/5/2021	100	100	100	0	Retained	High-quality
47	5/20/2021	96.4	97.6	97.6	2.4	Retained	High-quality

48	5/23/2021	100	100	100	0	Retained	High-quality
49	5/6/2021	100	97.3	95.9	4.1	Retained	High-quality
50	5/22/2021	100	100	95.7	4.3	Retained	High-quality
51	5/27/2021	97.4	92.1	92.1	5.3	Retained	High-quality
52	6/10/2021	85	88.3	90	5.8	Retained	High-quality
53	5/20/2021	98.1	100	100	0	Retained	High-quality
54	5/27/2021	96.7	80.3	77	24.6	Culled	Underperforming
55	5/27/2021	97.9	97.9	95.7	2.1	Retained	High-quality
56	5/26/2021	91.4	91.4	91.4	11.4	Retained	Underperforming
57	5/12/2021	100	100	94.1	5.9	Retained	High-quality
58	5/6/2021	98.7	97.4	98.7	2.6	Retained	High-quality
59	5/4/2021	91.7	83.3	83.3	16.7	Retained	Underperforming
60	5/18/2021	100	88.9	88.9	0	Retained	High-quality
61	5/6/2021	100	100	95.7	4.3	Retained	High-quality
62	3/12/2021	100	100	96.4	0	Retained	High-quality
63	5/12/2021	94.1	100	100	0	Retained	High-quality
64	5/27/2021	100	97.8	97.8	2.2	Retained	High-quality
65	5/20/2021	87.1	87.1	83.9	12.9	Retained	Underperforming
66	5/6/2021	100	95	100	5	Retained	High-quality
67	5/6/2021	100	94.7	100	5.3	Retained	High-quality
68	5/6/2021	100	100	100	0	Retained	High-quality
69	5/27/2021	91.7	91.7	91.7	11.1	Retained	Underperforming
70	6/2/2021	95	70	70	30	Retained	Underperforming
71	4/29/2021	96.4	92.9	92.9	7.1	Retained	High-quality
72	5/28/2021	100	74.2	71	29	Culled	Underperforming
73	6/2/2021	97.9	95.7	95.7	16.4	Retained	Underperforming
74	5/8/2021	82.4	58.8	41.2	64.7	Culled	Underperforming
75	6/9/2021	100	100	100	0	Retained	High-quality
76	5/30/2021	86.7	86.7	86.7	13.3	Retained	Underperforming
77	6/9/2021	94.4	94.4	88.9	11.1	Retained	Underperforming
78	5/6/2021	100	100	95.5	4.5	Retained	High-quality
79	5/27/2021	100	100	100	0	Retained	High-quality
80	5/20/2021	85.7	85.7	85.7	17.1	Retained	Underperforming
81	5/20/2021	100	100	100	0	Retained	High-quality
82	5/20/2021	100	100	96.8	3.2	Retained	High-quality
83	5/8/2021	88.9	100	100	0	Retained	High-quality
84	6/2/2021	100	100	100	0	Retained	High-quality
85	6/7/2021	86.7	40	60	60	Culled	Underperforming
86	5/20/2021	88.2	88.2	88.2	11.8	Retained	Underperforming
87	5/13/2021	94.4	88.9	88.9	11.1	Retained	Underperforming
88	5/9/2021	100	100	100	0	Retained	High-quality
89	5/13/2021	100	94.4	94.4	5.6	Retained	High-quality
90	5/13/2021	100	96.8	96.8	0	Retained	High-quality
91	4/26/2021	91.7	100	100	0	Retained	High-quality
92	5/6/2021	100	100	88.9	9.1	Retained	High-quality
93	5/6/2021	100	100	100	0	Retained	High-quality
94	6/2/2021	100	100	87.5	8.5	Retained	High-quality
95	4/8/2021	93.1	96.6	93.1	6.9	Retained	High-quality
96	5/6/2021	75	100	75	8	Retained	High-quality
97	5/6/2021	94.4	100	100	0	Retained	High-quality
98	5/8/2021	80	60	60	45	Retained	Underperforming
99	6/2/2021	95.5	95.5	95.5	4.5	Retained	High-quality
100	5/13/2021	82.4	76.5	70.6	35.3	Culled	Underperforming

101	5/20/2021	93.3	88.9	88.9	9.1	Retained	High-quality
102	6/9/2021	100	100	100	0	Retained	High-quality
103	5/13/2021	75	50	33.3	75	Retained	Underperforming
104	5/27/2021	75	87.5	87.5	8.5	Retained	High-quality
105	5/13/2021	76.5	64.7	52.9	58.8	Culled	Underperforming
106	5/27/2021	20	0	0	100	Retained	Underperforming
107	5/27/2021	0	0	0	100	Culled	Underperforming

[1] ID refers to the unique identifier of each boar, corresponding to the Boar ID shown in Figure 4.

[2] Pass Sperm Number (%) represents the percentage of valid samples for each boar in which total sperm count met the threshold ($\geq 20 \times 10^9$), as defined in Figure 3.

[3] Pass Motility (%) represents the percentage of valid samples for each boar in which motility score exceeded 70%, as defined in Figure 3.

[4] Pass Morphology (%) represents the percentage of valid samples for each boar in which morphology score exceeded 70%, as defined in Figure 3.

[5] Discard Rate (%) indicates the percentage of samples that were manually flagged as trash.

[6] Culling indicates whether a boar was culled from the breeding population due to poor semen quality. Such boars were directly classified as underperforming.
